# Risks of Myrrh usage in pregnancy

**DOI:** 10.5935/1518-0557.20160050

**Published:** 2016

**Authors:** Dania Al-Jaroudi, Ouhoud Kaddour, Nahla Al-Amin

**Affiliations:** 1 Department of Obstetrics and Gynecology, Reproductive Endocrine and Infertility Medicine Department, King Fahad Medical City (KFMC), Riyadh, Saudi Arabia; 2 Research Center, King Fahad Medical City (KFMC), Riyadh, Saudi Arabia

**Keywords:** Pregnancy, myrrh, recurrent pregnancy loss, Saudi Arabia, herbal medicine, commiphora

## Abstract

Recurrent miscarriage places a huge psychological burden on a woman who is trying to conceive. Meanwhile, the use of traditional medicine still plays an important role within the Saudi Arabian culture, where many patients still seek alternative forms of therapy. However, this traditional way of treatment might expose patients to many hazards. We present a case of a 32-year-old pregnant woman with a history of infertility and recurrent miscarriages. She used large amounts of myrrh herbs for 2 months, since a traditional healer told her that her current pregnancy would progress safely by its use. However, her pregnancy was complicated with an acute abdominal pain. Her symptom was relieved as soon as she stopped taking myrrh. We assume that myrrh acted as a uterine stimulant causing acute abdominal pain. Scientific studies should be carried out to evaluate the safety of Myrrh intake during pregnancy.

## INTRODUCTION

Traditional medicine in Saudi Arabia, based on herbal remedies and spiritual healing, still plays a prime role within the local Saudi culture. Some people strongly believe in the essential role of herbs for the treatment of medical conditions, including recurrent miscarriages, which are difficult to be treated in any way. One of the most used herbs in the Arabian Peninsula is called Myrrh, scientifically known as Commiphora Myrrh (El Ashry *et al*., 2003).

The Myrrh genus, Commiphora, contains approximately 190 species of shrubs and trees, which is distributed throughout the sub-tropical regions of Africa, the western Indian Ocean islands, the Arabian Peninsula, India, Vietnam, and South America (Nomicos, 2007). It may also be known as Murrah, Abyssinian Myrrh found in Ethiopia and Arabian Myrrh (El Ashry *et al*., 2003).

The composition of Myrrh includes a volatile essential oil, sesquiterpenes, which is an alcohol-soluble resin containing commiphoric acids and a water-soluble gum (Mills & Bone, 2005).

According to the Encyclopedia of Islamic Herbal Medicine (Goodier, 2012) "The Messenger of Allah stated: 'Fumigate your houses with al-shih, murr, and sa'tar'". The author claims that this use of the word "murr" refers specifically to Commiphora myrrha.

A study carried out by Chinese researchers, also found that resins from myrrh extracts might be effective against human gynecologic cancer cells (Su *et al*., 2011). Some women use myrrh oil during childbirth in order to lessen labor pain, an old practice that was exclusive for royalty (Hillson, 1988). The appropriate dose depends on several factors, such as the user's age, health, and several other conditions (El Ashry *et al*., 2003).

Large doses may be unsafe; amounts greater than 2-4 grams can cause kidney irritation and heart rate changes (El Ashry *et al*., 2003). There is not enough scientific information to establish an appropriate dose range for myrrh (El Ashry *et al*., 2003).

In terms of pregnancy and lactation, taking myrrh orally during pregnancy is unsafe and should be avoided. Myrrh can stimulate the uterus and might cause miscarriage or preterm labor; nevertheless, there isn't enough information to rate the safety of using myrrh locally on skin during pregnancy (Al Awadi & Gumaa, 1987). Breast-feeding women should also avoid using myrrh, since the safety of using it when breast-feeding is still unknown (Al Awadi & Gumaa, 1987).

Myrrh also stimulates uterine bleeding, which is why it is used by some women to enhance blood flow during the early days of their periods (El Ashry *et al.*, 2003). Taking Myrrh with Warfarin, an anticoagulant agent, decreases its action and increases the chance of blood clotting (Ernst, 2002).

## CASE REPORT

We present a case of a 32 years old pregnant Saudi woman, a current IVF pregnancy, with gestational age of 9 weeks. She had had a history of infertility for 10 years and a history of 4 previous spontaneous miscarriages despite thromboprophylaxis. The patient presented to the Emergency Room complaining of severe lower abdominal pain, worse on the right lower quadrant radiating to the right groin. It was associated with nausea and vomiting for 2 days. Upon examination, the patient was in pain, but vitally stable. Abdominal examination showed generalized lower abdominal tenderness, with positive rebound tenderness and positive renal angle tenderness over the right side as well.

Investigations included initial blood tests: complete blood count, renal function test, electrolytes, liver function test and serology, midstream specimen urine (MSU) analysis and all were found to be normal. Gynecological US confirmed the pregnancy by visualizing an intrauterine single viable embryo at gestational age of 8 weeks and 6 days, consistent with the Embryo Transfer (ET) date. The right ovary measured 89x47x61 mm with about 15 follicles, and the largest was 22x20 mm and the left ovary measured 68x45x52 mm with about 10 follicles, with the largest being 22x20 mm. Small volume of free fluid was seen in the pelvis. Ultrasound of the kidney, urethra, bladder, liver and biliary tract were normal.

After an initial denial of any history of herbal medicine intake, the patient later stated having 2 cups of myrrh a day. The Myrrh resin was dissolved in a 500 ml water bottle after consulting a traditional healer. The traditional healer advised the intake of myrrh to avoid pregnancy loss, which the patient complied with as soon as she discovered her pregnancy. In the hospital, after admission, the patient was advised to stop taking myrrh immediately and continue on enoxaparin 60 mg subcutaneous once a day. One day after doing so, the patient was discharged home as she was in a stable clinical condition, with no active complaints. She was followed throughout her pregnancy and delivered a normal healthy baby at 38 weeks of gestation. Patient's consent was taken for reporting her case and ethics' committee's approval was obtained through the Institutional Review Board to issue this case report.

## DISCUSSION

The C. molmol extract (myrrh) carries analgesic, anti-inflammatory and anti-hyperlipidemic effects (Chevallier, 1996). The main Myrrh components are: volatile oil (primarily sesquiterpenes), triterpenes, and gum resin with reported actions as analgesic, antifungal, antiseptic, astringent, carminative, emmenagogue, expectorant, antispasmodic, disinfectant, immune stimulant, circulatory stimulant, stomachic, tonic, and vulnerary (Chevallier, 1996).

The oily gum resin of Commiphora increases the number of leucocytes and stimulates phagocytosis and thus Myrrh is used for the treatment of the female reproductive tract disorders mainly as a disinfectant, and as an astringent (Chevallier, 1996, Saeed & Sabir, 2004).

This resin is the Myrrh component that acts as a uterine stimulant and emmenagogue (Saeed & Sabir, 2004). Despite its widespread usage, Myrrh is not recommended for pregnant women because of its effects as a uterine irritant (Chevallier, 1996, Saeed & Sabir, 2004). This is in agreement with to what has possibly happened to our patient, who had been admitted with acute abdominal pain, and whose symptoms were relieved as she stopped taking Myrrh.

Despite several reports on the effective usage of Myrrh, more evidence is needed to rate its actions, especially during pregnancy.

## CONCLUSION

While traditional healers would claim their curing abilities, the safety of herbal remedies has been always a subject for debate. Further scientific studies should be conducted to evaluate the safety of commonly used local herbal remedies, especially during pregnancy.

Until such conclusive results are available in the literature, the huge task of healthcare professionals is to educate the population and the healthcare professionals in order to gradually shift community beliefs from relying on spiritual healers with their herbal treatment of unknown safety to well recognized contemporary medications.
